# Cardiovascular events in crush syndrome: on-site therapeutic strategies and pharmacological investigations

**DOI:** 10.3389/fphar.2024.1472971

**Published:** 2024-09-20

**Authors:** Meng-Wan Zhang, Fu-Qin Tan, Jia-Rong Yang, Jian-Guang Yu

**Affiliations:** Department of Pharmacy, Shanghai Chest Hospital, School of Medicine, Shanghai Jiao Tong University, Shanghai, China

**Keywords:** crush syndrome, fluid resuscitation, hypovolemic shock, hyperkalemia, ischemia/reperfusion injury

## Abstract

Crush syndrome often occurs after severe crush injury caused by disasters or accidents, and is associated with high mortality and poor prognosis. Cardiovascular complications, such as cardiac arrest, hypovolemic shock, and hyperkalemia-related cardiac dysfunction, are the primary causes of on-site death in crush syndrome. Prehospital evaluation, together with timely and correct treatment, is of great benefit to crush syndrome patients, which is difficult in most cases due to limited conditions. Based on current data and studies, early fluid resuscitation remains the most important on-site treatment for crush syndrome. Novel solutions and drugs used in fluid resuscitation have been investigated for their effectiveness and benefits. Several drugs have proven effective for the prevention or treatment of cardiovascular complications in crush syndrome, such as hypovolemic shock, hyperkalemia-induced cardiac complications, myocardial ischemia/reperfusion injury, ventricular dysfunction, and coagulation disorder experimentally. Moreover, these drugs are beneficial for other complications of crush syndrome, such as renal dysfunction. In this review, we will summarize the existing on-site treatments for crush syndrome and discuss the potential pharmacological interventions for cardiovascular complications to provide clues for clinical therapy of crush syndrome.

## Introduction

Crush syndrome is usually caused by severe natural or human-made disasters, such as earthquakes, landslides, terrorist attacks, and air or railway crashes, and is characterized by systemic manifestations of traumatic muscle injury and subsequent multi-organ dysfunction or failure (Long et al., 2023; Usuda et al., 2023). Data based on current disaster investigations indicate that almost half of the survivors have been subjected to severe crush injuries, among which as high as 40%–70% suffered from crush syndrome (Long et al., 2023; Sever et al., 2012; Sever and Vanholder, 2013; Gibney et al., 2014). Crush syndrome can be complicated with hypovolemic shock, hyperkalemia, cardiac arrest, acute kidney injury, systemic inflammation, and sepsis, resulting in high mortality up to 48% (Sahjian and Frakes, 2007; Long et al., 2023; Usuda et al., 2023; Genthon and Wilcox, 2014; Metzger et al., 2010; McCullough et al., 2014; Sever et al., 2003). It is often difficult to accurately diagnose crush syndrome and initiate appropriate treatment in on-site situations (Usuda et al., 2023). As reported, approximately 20% of crush victims die of cardiac arrest induced by hyperkalemia or hypovolemic shock within a short time after decompression (Ashkenazi et al., 2005). A previous study also indicated that the survival rate of traumatic cardiac arrest patients with crush injuries was only 10.6% (Yang et al., 2022). Therefore, new therapeutic methods and effective drugs, especially those targeting cardiovascular symptoms in crush syndrome, are urgently needed. Recently, there have been some advances in the treatment and pathological mechanisms of crush syndrome, including novel therapeutic drug for crush-induced hyperkalemia and the role of ferroptosis in crush syndrome-related acute kidney injury (Li et al., 2024; Qiao et al., 2023). In this review, we make efforts to comprehensively summarize and illuminate current on-site therapies and pharmacological investigations of cardiovascular complications in crush syndrome. We also indicate the limitations and advantages of these potential drugs. For example, only a few drugs could be beneficial for multiple symptoms related to crush syndrome, whereas some drugs could treat hyperkalemia without the risk of hypokalemia in crush syndrome. Our findings provide insights for the future clinical treatment of crush syndrome.

## On-site therapeutic strategies

### Routine on-site treatments for crush syndrome

Immediate and proper on-site treatments are crucial for victims exposed to severe crush injuries to prevent potential crush syndrome or reduce the mortality of crush syndrome that already existed. As reported, even simple on-site therapies could reduce 13%–40% of early deaths (Long et al., 2023). With the exception of extrication as soon as possible, other effective treatments at the scene of the incident include right triage, airway support management with portable ventilators (especially for those chest crush victims), hemorrhage control with tourniquet, fracture fixation, fluid resuscitation, hypothermia prevention, pain control, and efficient transport (Zhang et al., 2014; Long et al., 2023; Sever et al., 2012; Ashkenazi et al., 2005). On-site care providers should especially notice the occurrence of hyperkalemia and inducible cardiac dysrhythmias by monitoring serum potassium levels and electrocardiographic parameters as early as possible and throughout the entire prehospital care period of crush injury patients to prevent sudden cardiac arrest or heart failure (Usuda et al., 2023; Genthon and Wilcox, 2014; Gonzalez, 2005; Burns et al., 2010).

Severe crush injury with serious muscular damage and large amounts of bleeding often leads to crush syndrome with multiple complications, such as traumatic rhabdomyolysis, acute compartment syndrome, acute kidney injury, and many cardiovascular-related symptoms, including cardiac or hypovolemic shock, metabolic acidosis, hyperkalemia, and disseminated intravascular coagulation (Sahjian and Frakes, 2007; Long et al., 2023; Usuda et al., 2023; Abu-Zidan et al., 2023). Hemorrhage-induced volume loss and fast transfer of extracellular fluids into injured tissues lead to hypovolemia during severe crush injury. Hypoperfusion of organs induces toxicity aggregation, resulting in severe complications of multiple systems in crush syndrome. For example, the release of myoglobin and toxic metabolites from damaged tissues along with renal hypoperfusion may facilitate renal tubular cast formation and finally cause acute renal failure. Early aggressive fluid resuscitation has been confirmed as the most essential on-site treatment to restore circulating blood volume and reduce complications from crush syndrome or even prevent rapid death after decompression (Sahjian and Frakes, 2007; Long et al., 2023; Usuda et al., 2023; Li et al., 2009). Sufficient fluid resuscitation could correct hypotension and acidosis, alleviate hypovolemia and hyperkalemia, increase urine flow to excrete toxins, relieve limb swelling, restore end-organ perfusion, and prevent heart or renal failure (Sahjian and Frakes, 2007; Long et al., 2023; Li et al., 2009).

### On-site fluid resuscitation in crush syndrome

Initiation of fluid resuscitation should be as early as possible even before extrication, best within 6 h of crush injury to reduce the risk of hypovolemic shock and acute kidney injury, and throughout the rescue process (Long et al., 2023; Usuda et al., 2023; Sever et al., 2012; Better and Stein, 1990; Better, 1990). The vascular access choice and volume of fluid resuscitation depend on victim’s status and environmental conditions, such as age, comorbidities, injury pattern, and crush duration (Abu-Zidan et al., 2023). Intravenous access is preferred at an earliest rate of 1–1.5 L/h for adults and 10–20 mL/kg/h for children if the extremity vein can be found, and the volume can be up to 6–12 L/d (Long et al., 2023; Genthon and Wilcox, 2014; Ashkenazi et al., 2005; Gonzalez, 2005; Sever et al., 2012). If not available, intraosseous or subcutaneous access is recommended for fluid resuscitation (Sahjian and Frakes, 2007; Sever et al., 2012; Sever and Vanholder, 2013; Gibney et al., 2014; Abu-Zidan et al., 2023). A urinary catheter could be needed to track urine output and a target urine output of more than 300 mL/h helps to prevent crush-induced acute kidney injury (Malinoski et al., 2004; Long et al., 2023; Scharman and Troutman, 2013; Sever et al., 2006).

Multiple fluids or drugs can be useful in fluid resuscitation processes, such as isotonic sodium chloride solution, 5% glucose, blood or artificial plasma, sodium bicarbonate, 20% mannitol, human serum albumin, 5% dextran, and diuretics, when circulation is steady (Sahjian and Frakes, 2007; Long et al., 2023; Genthon and Wilcox, 2014; Li et al., 2009). Potassium-containing solutions such as Ringer’s lactate should be avoided for crush patients during fluid resuscitation in cases of hyperkalemia which could cause cardiac dysrhythmias (Sever and Vanholder, 2013). Doctors should properly make decisions about the single, combined, or sequential utilization of these fluids according to the patient’s situation. A variety of studies have proven that the first-line recommendation of fluid resuscitation for crushed patients is isotonic saline, which is most effective for volume replacement, to correct hypotension or tachycardia, prevent hypovolemic shock, reduce acute kidney injury and the use of renal replacement therapy, and avoid death (Long et al., 2023; Walters et al., 2016; Sahjian and Frakes, 2007; Gunal et al., 2004; Genthon and Wilcox, 2014). In some cases, blood products can be used to treat hypotension or coagulopathy by transfusing plasma or platelets to supplement essential blood components in time (Sahjian and Frakes, 2007; Li et al., 2009; Sever et al., 2012; Walters et al., 2016).

It is also recommended that sodium bicarbonate be added to fluids to reduce metabolic acidosis, treat hyperkalemia, and alkalinize the urine (Sahjian and Frakes, 2007; Better, 1999; Long et al., 2023; Usuda et al., 2023). In crush syndrome, severe acidosis damages cardiac function by decreasing myocardial contractility and cardiac output and causing arrhythmias or hypotension (Sabatini and Kurtzman, 2009; Kraut and Kurtz, 2006; Kraut and Madias, 2010). Breakdown of myoglobin from injured muscles may cause tubular epithelium slough and cast formation in the kidney, obstruction of renal blood flow, and finally, renal failure. Alkalinization of the blood and urine by sodium bicarbonate could reduce the harmful effects on the cardiovascular system and myoglobin breakdown by promoting the precipitation of myoglobin (Sahjian and Frakes, 2007; Criddle, 2003; Long et al., 2023; Genthon and Wilcox, 2014). The therapeutic goal of urine alkalinization is to achieve a pH of 6–7 (Sahjian and Frakes, 2007; Genthon and Wilcox, 2014). Sodium bicarbonate also has a temporary effect on treating hyperkalemia, although this impact has been doubtful in the absence of acidemia (Long et al., 2018; Weisberg, 2008; Allon and Shanklin, 1996).

A 20% mannitol solution is recommended after adequate resuscitation and urine output (Smith and Greaves, 2003; Sever et al., 2006; Sahjian and Frakes, 2007; Gonzalez, 2005). Mannitol is mainly used to force diuresis, clear myoglobin, scavenge free radicals to avoid oxidative injury, and reduce tissue swelling or alleviate compartment pressures to avoid compartment syndrome (Sahjian and Frakes, 2007; Malinoski et al., 2004; Genthon and Wilcox, 2014). With a urine flow of more than 20 mL/h, mannitol can be added at a total dose of 1–2 g/kg/d at an infusion rate of 5 g/h (Sever et al., 2006). It is worth noting that anuric patients or those who have been volume-depleted should not use mannitol. Other contraindications for mannitol include congestive heart failure and electrolyte abnormalities (Smith and Greaves, 2003; Sever et al., 2012; Gibney et al., 2014). Monitoring electrolyte disturbances and serum osmolality when mannitol is infused is necessary, in order to avoid hypokalemia which may cause cardiac dysrhythmias or arrest and even death, and to avoid an increase in osmolality which might indicate a dehydrated situation (Sahjian and Frakes, 2007). Although mannitol and bicarbonate (even the cocktail administration of mannitol-bicarbonate, with a mix of bicarbonate, mannitol, sodium chloride, and dextrose) are believed to protect against acute renal failure in crush syndrome, reliable data from randomized controlled trials are still needed to confirm the exact effects of bicarbonate and/or mannitol for fluid resuscitation in crush syndrome (Altintepe et al., 2007; Chavez et al., 2016; Schwartz et al., 2015; Brown et al., 2004).

## Pharmacological investigations of cardiovascular complications

### Hypovolemic shock in crush syndrome

Hypovolemic shock is one of the most common complications in crush syndrome. Lower cardiac output, sharply decreased mean arterial blood pressure, and increased heart rate usually appear in crush syndrome patients with hypovolemic shock (Usuda et al., 2023; Emig et al., 2003; Xu et al., 2018). Hypovolemic shock in crush syndrome is mainly caused by excessive hemorrhage-induced severe circulatory volume inadequacy and tissue hypoperfusion as well as consequent microcirculation disturbances and other metabolic alterations. Myoglobin released from damaged skeletal muscle into the circulation can induce vasoconstriction in multiple organs, leading to increased vascular resistance and an obstacle for sufficient blood supply to tissues in crush syndrome-related hypovolemic shock (Emig et al., 2003). Currently, sufficient fluid resuscitation and suitable liquid selection are still the most important methods for preventing and treating hypovolemic shock in crush syndrome, and more helpful candidate drugs are being researched.

Potential resuscitation solutions have been studied for their effectiveness and benefits compared with conventional fluid resuscitation (Song et al., 2013; Katz et al., 2002; Philbin et al., 2005; Kaplan et al., 2006; Malkevich et al., 2008; Mohri et al., 2006). Researchers found that in comparison with hypertonic or normal saline, bolus infusion of hypertonic hydroxyethyl starches (HHESs) increased the survival rate of crush-injured rats 72 h after decompression and their mean arterial blood pressures, and also improved the acid-base balance and renal function, proving that HHES combined with normal saline might be a better choice for fluid resuscitation to treat hypovolemic shock in crush syndrome (Song et al., 2013). Even so, investigations on hemoglobin-based oxygen carrier (HBOC-201), a bovine hemoglobin solution that improves oxygen delivery to tissues without obvious vasoconstriction, have demonstrated that HBOC-201 could be superior to HHES in hypovolemic shock in a crush-induced hemorrhagic shock model. Compared with HHES, resuscitation with HBOC-201 resulted in significantly higher survival rates, mean arterial blood pressures, mean pulmonary artery pressures, and pulmonary capillary wedge pressures, lower heart rates and lactate levels, and better cardiac index in crush-injured models. Animals treated with HBOC-201 also had higher tissue oxygenation and strong ion differences, as well as a lower need for resuscitated fluids or blood transfusion (Katz et al., 2002; Philbin et al., 2005; Kaplan et al., 2006; York et al., 2003; McNeil et al., 2001; Sampson et al., 2003; Knudson et al., 2003; Fitzpatrick et al., 2004; Lee et al., 2002). In addition, the combined use of HBOC-201 and recombinant factor VIIa during fluid resuscitation helped reduce immune activation in pigs with crush-induced hemorrhagic shock (Malkevich et al., 2008). Administration of recombinant human soluble thrombomodulin with fluid resuscitation increased the survival rate of crush-injured rats by attenuating hemoconcentration, manifested as increased hemoglobin and hematocrit levels, mitigating hyperkalemia, decreasing serum interleukin-6 and lactate levels, and improving acid-base balance and renal function (Mohri et al., 2006). These potential solutions or additive drugs for fluid resuscitation exert better anti-shock effects by elevating mean arterial blood pressures, regulating pH or lactate levels of blood, improving cardiac function, inhibiting immune responses, reducing blood creatinine and K^+^ levels, and mitigating hemoconcentration in crush syndrome.

**FIGURE 1:**
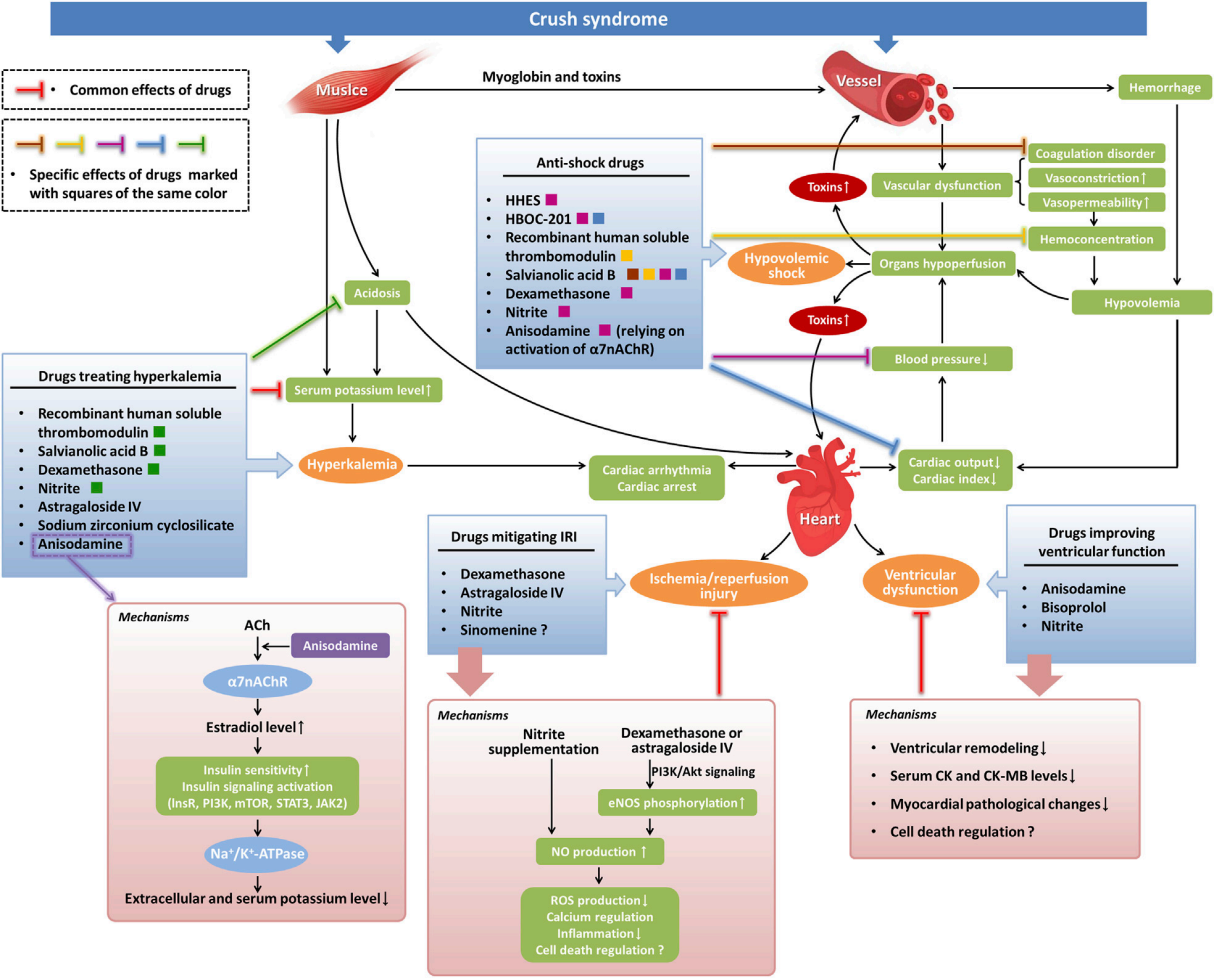
Potential pharmacological investigations of cardiovascular complications in crush syndrome. Severe crush injuries of muscle lead to the release of myoglobin and toxic cellular components from damaged cells to the blood in crush syndrome, resulting in vascular dysfunction, such as the occurrence of endothelial inflammation-induced coagulation disorder and an increase in vasoconstriction and vasopermeability. Large amounts of bleeding, combined with high vasopermeability-induced hemoconcentration, cause hypovolemia in crush syndrome, which leads to hypotension by reducing cardiac output. Hypoperfusion of vital organs induced by hypovolemia, hypotension, and vascular dysfunction in crush syndrome causes hypovolemic shock and increases toxic effects in tissues. Muscle damage-induced and acidosis-induced hyperkalemia in crush syndrome can lead to cardiac arrhythmia or even cardiac arrest. Crush syndrome can also cause myocardial IRI and ventricular dysfunction. In our findings, several anti-shock drugs exert their effects by alleviating coagulation disorders and decreasing hemoconcentration. Other anti-shock drugs treat hypovolemic shock by directly increasing blood pressure or by improving cardiac output. Drugs treating hyperkalemia function by reducing serum potassium levels directly or relying on the alleviation of acidosis. Drugs that mitigate myocardial IRI mainly depend on NO-mediated signaling pathways. The mechanisms involved in drugs improving ventricular function include attenuation of ventricular remodeling, decrease in serum CK and CK-MB levels, inhibition of myocardial pathological changes, and regulation of cell death possibly. ACh, acetylcholine; lnsR, insulin receptor; PI3K, phosphoinositide 3-kinase; mTOR, mammalian target of rapamycin; STAT3, signal transducer and activator of transcription 3; JAK2, Janus kinase 2; NO, nitric oxide; eNOS, endothelial nitric oxide synthase; ROS, reactive oxygen species.

In addition to the components used for fluid resuscitation, other drugs have been tested for their anti-shock effects in crush syndrome. Administration of salvianolic acid B or dexamethasone before fluid resuscitation could improve the survival rates in crush syndrome models through its anti-shock and anti-inflammatory effects, manifested as mean arterial blood pressure elevation, hematocrit level decrease, endothelial inflammation-induced coagulation disorder alleviation, acid-base balance enhancement, hyperkalemia control, renal function improvement, decrease in serum lactate and interleukin-6 levels, and inhibition of myeloperoxidase activity (Murata et al., 2022; Murata et al., 2016; Murata et al., 2013). Our previous studies also demonstrated the effects of anisodamine on crush syndrome-related shock by increasing blood pressure in crush syndrome rats, which relied on the activation of α7 nicotinic acetylcholine receptor (α7nAChR) (Yu et al., 2019; Fan et al., 2016) (Figure 1: Anti-shock drugs). Moreover, the anti-shock effect of anisodamine was strengthened by the co-administration of neostigmine in crush syndrome animals (Xu et al., 2016).

### Hyperkalemia-induced cardiac complications in crush syndrome

Several studies have reported that crush-injured patients or models can be complicated with hyperkalemia, cardiac arrest, arrhythmia, and heart failure (Yang et al., 2022; Whiffin et al., 2019; Guo et al., 2013; Murata et al., 2011; Safari et al., 2017; He et al., 2011; Fernandes et al., 2022; Yu et al., 2022; Murata et al., 2022). Hyperkalemia, with triggered cardiac or renal complications, is a major cause of death in crush syndrome. Severely increased potassium in the blood can lead to cardiac dysrhythmia, such as ventricular fibrillation, and even cardiac arrest without proper treatment in time, resulting in high mortality in crush syndrome (Metzger et al., 2010; McCullough et al., 2014). Potassium levels are also believed to be closely associated with mortality in acute myocardial infarction (McCullough et al., 2014; Goyal et al., 2012). Clinically, 70% of patients with hyperkalemia manifest typical electrocardiographic changes, such as elevated ST segments, tall-peaked T waves, PR interval lengthening, and P wave loss, as well as QRS complex widening. Acute treatments for hyperkalemia traditionally include intravenous calcium gluconate, correction of acidosis with sodium bicarbonate, and use of nebulized albuterol in some conditions (McCullough et al., 2014; Yu et al., 2022).

Currently, several novel drugs have been proven effective for hyperkalemia in crush syndrome and crush injury, consequently decreasing the on-site mortality. Co-administration of recombinant human soluble thrombomodulin or astragaloside IV with resuscitating fluids could decrease plasma potassium levels in crush-injured rats (Mohri et al., 2006; Murata et al., 2017). Dexamethasone treatment in the prehospital period also attenuated hyperkalemia in crush syndrome (Murata et al., 2016; Murata et al., 2013). Salvianolic acid B reduced plasma potassium levels, and improved the survival rate by alleviating hyperkalemia-induced cardiac failure, regulating acid-base balance, improving mitochondrial function, and decreasing the inflammatory response (Murata et al., 2022). Early intervention with sodium zirconium cyclosilicate reduced blood potassium levels in a rat crush syndrome model with no adverse effect on renal function (Li et al., 2024). Our studies demonstrated that anisodamine could alleviate hyperkalemia in crush syndrome animals with no risk of hypokalemia. Although the mechanisms involved in the downregulation of blood potassium levels for some drugs are still unclear, we found some clues regarding the effects of anisodamine. Anisodamine could decrease serum potassium levels in crush syndrome animals through activation of α7nAChR and downstream estradiol-induced enhancement of insulin sensitivity, manifested as an increase in serum estradiol which leads to the activation of tyrosine kinase on the insulin receptor, phosphoinositide 3-kinase, mammalian target of rapamycin, signal transducer and activator of transcription 3, and Na^+^/K^+^-ATPase (Yu et al., 2019; Fan et al., 2016) (Figure 1: Drugs treating hyperkalemia). Both the anti-hyperkalemia and anti-shock effects of anisodamine contribute to the reduction in the on-site mortality of crush syndrome animals. Another investigation on the combined use of anisodamine and neostigmine also demonstrated their effect on serum potassium decrease in crush syndrome, which depended on the role of α7nAChR in increasing the phosphorylation of Janus kinase two and signal transducer and activator of transcription 3 (Xu et al., 2016).

### Other cardiovascular complications in crush syndrome

In addition to shock- and hyperkalemia-induced cardiac complications, crush syndrome can also induce other cardiovascular injuries and impairment of cardiac function, such as myocardial ischemia/reperfusion injury (IRI), ventricular dysfunction, aortic injury, and venous thromboembolic disease (Adachi et al., 1996; Simpson et al., 2023; Mosquera et al., 2013; Liu et al., 2013; Lopez et al., 2021; Rawlins et al., 1999; Hoofnagle et al., 2023; Yu et al., 2022; Murata et al., 2022; Odeh, 1991; Kobayashi and Murata, 2018; Murata et al., 2012). Bisoprolol, a selective β1-blocker, used in combination with saline resuscitation alleviated the changes in the myocardial enzymes creatine kinase (CK) and creatine kinase-MB (CK-MB) in serum and the pathological changes in myocardial tissues in crush-injured rats. It also relieved ventricular remodeling, exhibiting a reduction in left ventricular mass, left ventricular volume at end-systole, and left ventricular internal diameter. The protective role of bisoprolol against heart injury in crush syndrome might result from its effects on decreasing the demand for oxygen and nutrients in the heart and reducing the toxicity of excessive catecholamines on cardiac myocytes, depending on selective and competitive blockade of β1 adrenergic receptors. These effects of bisoprolol led to an increase in the survival rate of crush-injured rats (Yu et al., 2022). Interestingly, our previous study found that anisodamine could also reduce CK and CK-MB levels in the serum of rats with crush syndrome (Fan et al., 2016) (Figure 1: Drugs improving ventricular function). Moreover, it has been reported that in crush injury, oscillatory shear stress-induced gene expression of FOXC2 and PROX1 could regulate the crush-induced procoagulant phenotype and the deep venous thrombosis-related valvular phenotype, guiding us in a novel direction towards crush syndrome therapy (Hoofnagle et al., 2023).

Crush syndrome is usually characterized by IRI (Odeh, 1991; Kobayashi and Murata, 2018). Throughout compression and subsequent rescue and therapy, multiple vital organs, such as the heart, can be damaged during both ischemia and reperfusion periods (Odeh, 1991; Kobayashi and Murata, 2018; Jennische, 1984; Korthuis et al., 1989; Sexton et al., 1990). The mechanisms underlying myocardial IRI in crush syndrome may involve oxidative stress, calcium regulation, and leukocyte activation (Odeh, 1991; Watts et al., 1980; Allen and Orchard, 1987). Specific therapeutic methods for myocardial IRI in crush syndrome are lacking, although there has been a report mentioning tourniquet application to attenuate IRI in crush injury (Schwartz et al., 2015).

Nitrite has been thought beneficial for the treatment of IRI experimentally (Kobayashi and Murata, 2018; Murata et al., 2012). Nitrite deficiency may cause severe myocardial dysfunction such as IRI-induced myocardial infarction, which can be relieved by nitrite supplementation (Bryan et al., 2007). Administration of nitrite before reperfusion in crush-injured rats covered the deficit of cardiac and systemic nitrite and restored nitric oxide bioavailability, leading to the alleviation of increased potassium, lactate dehydrogenase, and CK, inhibition of systemic inflammation, and reduction of crush-injured rat mortality (Murata et al., 2012). The underlying mechanisms by which nitrite alleviates myocardial IRI in crush syndrome might be related to nitric oxide-mediated cytoprotection, such as the reduction of reactive oxygen species production, regulation of Ca^2+^ channels, and attenuation of systemic inflammation (Chouchani et al., 2013; Sun et al., 2007; Sun and Murphy, 2010). In addition, the therapeutic effects of dexamethasone and astragaloside IV on crush syndrome include the prevention of IRI by elevating nitrite levels, increasing nitric oxide production via the phosphoinositide 3-kinase/Akt signaling pathway, and enhancing endothelial nitric oxide synthase phosphorylation (Murata et al., 2013; Murata et al., 2017; Kobayashi and Murata, 2018) (Figure 1: Drugs mitigating IRI). Studies have reported that sinomenine can attenuate cerebral, renal and hepatic IRI, as well as myocardial IRI (Yang et al., 2016; Zhao et al., 2013; Song et al., 2010; Lu et al., 2022; Xia et al., 2022). Sinomenine may be a promising drug for myocardial IRI therapy in crush syndrome, which requires further research (Zhang et al., 2021). In addition, several regulated cell death patterns, such as apoptosis and ferroptosis, are involved in crush syndrome and its complications. Cardiovascular complications in crush syndrome may be related to the death of cardiovascular cells (Liu et al., 2013; Qiao et al., 2023; Usuda et al., 2023). Studies focusing on death regulation in cardiomyocytes and vascular cells may also be beneficial for therapeutic strategies of crush syndrome in the future.

Early and proper pharmacological therapy is important for the treatment of crush syndrome. Besides making efforts to reduce on-site mortality by modulating cardiovascular symptoms, treatment of other systemic complications is also necessary. For example, anisodamine and salvianolic acid B can protect both the cardiovascular system and kidney function, manifested as a decrease in serum creatinine and blood urea nitrogen (Yu et al., 2019; Fan et al., 2016; Murata et al., 2022). Drugs with multiple therapeutic effects appear to be a promising strategy for treating crush syndrome. However, most of the potential drugs for crush syndrome included in our review are focused on the treatment of a single symptom or simplex systemic complications involved in crush syndrome, such as cardiovascular systemic symptoms. In addition, the cellular or molecular regulatory pathways of these therapeutic drugs have not been fully illustrated because the specific underlying mechanisms are not entirely clear. These limitations lied in our review call for further studies on these drugs.

## Conclusion

Current prehospital treatments for crush syndrome have focused on prompt fluid resuscitation and precautions against complications associated with crush syndrome, such as hyperkalemia, cardiac dysrhythmias, and acute kidney injury. Crush syndrome-related cardiovascular complications lead to a low survival rate and poor prognosis in crush syndrome patients, while safe and effective therapeutic methods are still lacking. Several drug candidates have proven promising for the treatment of hypovolemic shock, hyperkalemia-induced cardiac complications, myocardial IRI, ventricular dysfunction, and coagulation disorders, as summarized in Table 1. Further clinical investigations in patients with crush syndrome are required to promote the clinical application of these drugs in crush syndrome therapy.

**TABLE 1:** Drug candidates besides fluid resuscitation for cardiovascular complications in crush syndrome.

Candidate drugs	Cardiovascular effects	Other effects in crush syndrome	References
HHES	- increase mean arterial blood pressure	- improve acid-base balance - improve renal function	Song et al. (2013)
HBOC-201 (single use or combined use with recombinant factor VIIa)	- increase mean arterial blood pressure, mean pulmonary artery pressure and pulmonary capillary wedge pressure - decrease heart rate - improve cardiac index	- improve acid-base balance and decrease serum lactate level - increase tissue oxygenation - reduce fluid requirement and blood transfusion - reduce immune activation	Katz et al., 2002; Philbin et al., 2005; Kaplan et al., 2006; Malkevich et al., 2008; York et al., 2003; McNeil et al., 2001; Sampson et al., 2003; Knudson et al., 2003; Fitzpatrick et al., 2004; Lee et al., 2002
Recombinant human soluble thrombomodulin	- decrease hemoglobin and hematocrit levels - mitigate hyperkalemia	- improve acid-base balance and decrease serum lactate level - inhibit inflammatory response - improve renal function	Mohri et al. (2006)
Salvianolic acid B	- increase mean arterial blood pressure - mitigate hyperkalemia - decrease hematocrit level - reduce endothelial inflammation-induced coagulation disorder	- improve renal function - improve acid-base balance - improve mitochondrial function - inhibit inflammatory response	Murata et al. (2022)
Dexamethasone	- increase mean arterial blood pressure - mitigate hyperkalemia	- improve renal function - improve acid-base balance and decrease serum lactate level - inhibit inflammatory response	Murata et al., 2016; Murata et al., 2013
Anisodamine (single use or combined use with neostigmine)	- increase mean blood pressure, systolic blood pressure and diastolic blood pressure - mitigate hyperkalemia - decrease serum myocardial enzymes CK and CK-MB	- improve renal function - inhibit inflammatory response	Yu et al., 2019; Fan et al., 2016; Xu et al., 2016
Astragaloside IV	- mitigate hyperkalemia	- improve renal function - inhibit inflammatory response	Murata et al. (2017)
Sodium zirconium cyclosilicate	- mitigate hyperkalemia	—	Li et al. (2024)
Bisoprolol	- decrease serum myocardial enzymes CK and CK-MB - mitigate crush syndrome-induced myocardial pathological changes - relieve ventricular remodeling	—	Yu et al. (2022)
Nitrite	- increase mean arterial blood pressure - decrease serum myocardial enzyme CK - mitigate hyperkalemia	- improve acid-base balance - inhibit inflammatory response	Murata et al. (2012)
